# Obituary: Stefan Silbernagl — physiologist, gentleman, aesthete

**DOI:** 10.1007/s00424-025-03070-y

**Published:** 2025-02-28

**Authors:** Michael Gekle

**Affiliations:** Julius-Bernstein-Institute of Physiology, Martin Luther University Halle-Wittenberg, Magdeburger Strasse 6, Halle (Saale), 06112 Germany

If you wanted to have a witty conversation concerning driving skills in car rallies (preferably in a red Alfa Romeo), feature films from the golden era of Hollywood or Munich’s auteur cinema, the German post-war literature of Uwe Johnson, advanced black and white photographs by Ansel Adams, popular exhibitions of the fine arts, the inner workings of a model railway, red wine discoveries of the new world or perhaps “just” about the functioning of a mammalian kidney; when looking for suitable conversation partners for each of these topics, the answer was always: Stefan Silbernagl — physiologist, gentleman, aesthete.

## Early years — electrical engineering or medicine

His enthusiasm for technology and his technical talent were already evident in his youth. In particular, his fascination with complex mechanics and electronics. Therefore, the choice of course after graduating from high school was only logical: electrical engineering in 1958 in Munich. Although there was nothing wrong with the course in terms of subject matter, he had to realize that he was surrounded only and exclusively by men. A certain one-dimensionality, a lack of interest in aesthetic aspects, was noticeable and unavoidable, as he once described in a humorous mood. Perhaps without primarily intending it, he realized that the mix made the difference. He realized that purely male societies were missing something essential; he already had an eye on a topic that is present today. Although this was probably not the only reason, it was nonetheless not an unimportant one that prompted him to study human medicine at the LMU Munich in 1959 (and say goodbye to electrical engineering).

However, he did not abandon the “technical” aspect itself, but transferred it (today it is also called transdisciplinarity) into his approach to medicine. It was therefore no surprise that his foray into working as a general practitioner after completing his studies was short-lived and that in 1968 he began his career as a physiologist at the Physiological Institute of the LMU Munich.

## Physiology — research and recognition — the beauty in the complexity of kidney function

Physiology — “Der Gipfel der gesamten Naturwissenschaft und zugleich ihr dunkelstes Kapitel” (“The pinnacle of all natural science and at the same time its darkest chapter”; Arthur Schopenhauer: Gesammelte Briefe, Seite 296, Hg.: Arthur Hübscher; Bonn, Bauvier 1987) — enabled Stefan Silbernagl to combine medicine with technology in the sense of gaining knowledge, i.e., science. He devised precise questions (hypotheses), preferred complex experimental setups with control technology, and placed value on valid and precise measurements (the somewhat forgotten field of biological-medical measurement technology), mathematically correct evaluation and analysis as a counterpoint to clinical phenomenology. He wanted to understand mechanistically, to grasp connections and was aware that in the biomedical field, this usually resulted in complex, strenuous, non-linear principles. But it was precisely these principles that had to be used in order to outline or describe new paths.

It was a fortunate twist of fate that Stefan’s enthusiasm and talent met pressing, open questions concerning kidney function at the Munich (and later Innsbruck, Austria) Institute, since the kidney, after the brain, is probably the most complex organ. For many medical students, it is a multidimensional, non-linear nightmare of filters, tubes, transporters, driving forces, and control circuits (I am just saying countercurrent multiplication and tubulo-glomerular feedback).

For Stefan Silbernagl, the pivotal question was what happens to amino acids in a tube lined with cells with an inner diameter of only 10–20 µm — when this tube is still in the intact kidney, in the living organism (in situ et vivo). For the tinkerer and hobbyist, this was exactly the right challenge: precise measurements in nanoliter volumes of endogenous fluids (tubule fluid), using specially adapted liquid chromatography, among other things, to analyze and calculate dynamic variables (e.g., epithelial permeability and flux rates).

Did you know that we filter 50 g of amino acid per day in our kidneys? This is close to the minimum necessary daily supply (or almost half a quarterpounder)! Thus, the glomeruli are spendthrifts and the renal tubuli must prevent the worst from happening. But it was unknown how in those days. There are relatively few specific diseases, not because it is not important, but because it is so important that evolution did not tolerate dysfunction. Thus, fundamental knowledge was missing.

Physiology and pathophysiology of amino acid transport in the renal tubular system: important, complex, a rocky road, and apparently not very sexy. But someone has to do it. So he set out. Renal amino acid transport, when the renal world focused primarily on potassium, protons, glucose, urea, and ammonium.

Stefan accepted the challenge and worked consistently to elucidate the mechanisms that prevent our valuable amino acids from being lost with the urine. The fruits of his early work (e.g., [1, 5, 7, 8]), which was based primarily on micropuncture studies on intact tubules in vivo et situ (!), culminated in the groundbreaking review “The renal handling of amino acids and oligopeptides” [9]. He found support in 1976 by his “brother in spirit,” W.H. Dantzler from the University of Arizona, who was also an aficionado of the micropuncture technique and, together with Stefan and his basic stoicism, pushed it into the depths of the loop of Henle and the vasa recta [2]. This scientific relationship would turn into friendship and Bill and Stefan would spend several scientific periods together alternating between Germany and Arizona.

In 1981, Stefan Silbernagl was appointed full professor of physiology and head of the Institute of Physiology at the University of Würzburg, Germany. In Würzburg, he continued the micropuncture work, at the same time extending the experimental approach to new in situ and cell physiological models (e.g., [3, 4, 6]). Consequently, he got involved in nephrotoxicity as well as cellular calcium and proton homeostasis. Nevertheless, he remained faithful to his early “scientific love,” micropuncture in vivo et situ, until the end of his career. His last publication combines classical renal clearance with micropuncture studies on a topic of tubular amino acid transport under physiological and nephrotoxic conditions [6].

## Physiology — teaching and learning

Being a faculty member at a university meant for Stefan being researcher and teacher, at equal rights. Right from the beginning of his academic career, he was engaged in teaching per se and in the development of teaching. In his case, this did not mean to develop POL courses or inverted classrooms but to develop learning material of high profoundness and quality that at the same enables students to understand the complex principles of “The pinnacle of all natural science ….”, i.e. physiology. Together with Agamemnon Despopoulos and the Thieme Verlag, he developed the concept for the “Color Atlas of Physiology,” published first in 1979 and now in its 9th edition and translated in several languages (and apparently the most stolen textbook in medical libraries in Germany). They were aware of the fact that the demanding subject physiology, being quantitative, non-linear, mechanistic had to be presented in a different, modern way for new generations of students. Although traditionalist medical circles initially mocked the “comic of physiology,” in the end, the quality, rich content, and design concept prevailed. The mockers have since fallen silent and been converted.

I personally like the idea that this project also reveals the aesthete in Stefan Silbernagl. In 1994, Thieme Verlag convinced him to join the market of classical textbooks of physiology and so he became co-editor of “The Textbook of Physiology,” which is now in its 10th edition. Finally, in 1998, Stefan Silbernagl and Florian Lang started co-editing the “Color Atlas of Pathophysiology,” another success story of the new design concept.

## Academic mentor — personnel development

Personnel development was not at the center of academia or academic competence (and there is still room for improvement) in the second half of the last century. Yet, you could no longer linger under a tree with your teacher and indulge in gaining knowledge.

My relationship with Stefan Silbernagl started in 1986 as my doctoral supervisor. It was my third year in medical school and I had just moved to the University of Würzburg. Thus, I did not know any of the physiologists there in person. Nevertheless, from the experience of my first two years at medical school in Homburg (Universität des Saarlandes) it was clear to me that only physiology would be intellectually satisfactory (see A. Schopenhauer) for a doctoral thesis. So I went straight to the Institute of Physiology in Würzburg, knocked at his door and said “I want to do my doctorate here.” Few weeks later, I went again to his office and came back with some 20 papers (hard copy!) regarding the possibility of renal tubular amino acid secretion. “Read these publications, prepare an excerpt on the hints for secretion and ways to test this hypothesis,” was his assignment. It turned out that under certain conditions the quantitative comparison of renal excretion rates of certain amino acids with their calculated filtered amount inevitably led to the working hypothesis that net secretion (and thus basolateral uptake) of amino acids can occur. Starting from observations, a functional concept and some mathematics, a rational hypothesis developed. A certain stringency combined with aesthetics, just the way Stefan liked it — and I was probably just right for him. He then gave me publications on the structure of the amphibian kidney that might be helpful to proof secretion and a place in the laboratory. Initially, I had no idea but I did it anyway, in a very friendly and extremely supportive atmosphere in the institute. After 4 years, we knew there is tubular amino acid secretion in the intact kidney [3]. Few years later, molecular cloning of amino acid transporters by various other groups identified the underlying cellular mechanisms.

This brief personal introduction should help to characterize Stefan Silbernagl as an academic mentor. He was convinced that young academics must be granted freedom — within a certain corridor, or course. For mentors, granting this freedom means generating creative spaces (Gestaltungsräume) in which young academics can develop and distinguish themselves. This means that young scientists can take advantage of opportunities, but it also means that they can fail because of this freedom. From today’s perspective, in which mentoring is often confused with guarantees of success, this may sound unusual and would possibly be viewed negatively in line with the zeitgeist. However, a critically reflection would lead to a different assessment. Scientific work at a university means the ability to fill creative spaces independently and successfully. Another important aspect of Stefan’s mentoring was his ability of letting go and allowing success of others to happen. Young scientists should not strive for his fame, but fight for their own profile themselves. He had no problem to step aside.

Personnel development means not only mentoring of young scientists. Those who limit themselves to this will not be successful in the long term. The secret of long-term success in research and teaching is in technical and administrative science-supporting personnel. Stefan Silbernagl always communicated with the science-supporting staff as equals and took their concerns seriously. He taught all members of the institute to appreciate the “flat hierarchy” and its positive effect on the working atmosphere and staff motivation. He always had an open ear, even for seemingly “trivial” issues from the perspective of the academic ivory tower. However, there was one daily critical phase: after the lunch break, when Stefan took his sacred half-hour “power nap.” His secretary kept watch in the anteroom with a serious face and would not let anyone into his office, not even the dean.

## Manager — be friendly, do not make friend

Being a faculty member at a university meant for Stefan Silbernagl not only being researcher and teacher, but also to fulfil the obligations of academic self-administration. As it turned out, he had management talent well above the average professor. This turned out to be a great advantage for Würzburg University Medicine, as he proved to be a fair, impartial, and incorruptible manager and organizer. His unspoken credo was to be friendly but do not necessarily make friends. The systematic approach of Stefan (although his office with various stacks of paper on different tables did not look like that to the simple observer — chaos with hidden systematics) has also helped to shape the positive development of faculty structures and thus had a lasting impact. In this sense, he served as dean from 1987 to 1989 and from 2002 to 2004. Furthermore, he served as dean of study from 1996 to 2002. Especially the period from 1996 to 2004 was coined by changes of the traditional university culture, starting with the faculty self-administration of global budgets (give and take) through to the institutionalized evaluation of teaching staff by students. Fortunately, there was someone with management skills who did not hesitate making himself unpopular if it served the cause.

Stefan Silbernagl was also committed to the German Physiological Society and acted as Congress President in 1988, when the congress in Würzburg was organized jointly with The Physiological Society. A big congress for the small city of Würzburg that kept the entire institute busy for weeks. He later became involved in the Society’s public relations work and was editor of relevant publications. After retiring, he became an honorary member of the German Physiological Society.

Further evidence of his commitment to the academic landscape is his membership of the founding committee of the Faculty of Medicine at the Technical University of Dresden (1991–1994, when he also enjoyed opera, museums, and art in Dresden) and his role as spokesperson for the DFG Collaborative Research Centre 176 (1988–1999). A consistent feature of his commitment was his mode of work in the background. Visibility was no value per se for him, as it seems to be for so many today, but the unavoidable consequence of competence and quality.

## La vita e bella — his life was beautiful

Stefan was a bon vivant that enjoyed lobster as well as bratwurst. He loved red wine and, in earlier times, sometimes a nice cigar. However, as head of the institute, he never smoked in the laboratory. Typical for him, in order not to bother the laboratory staff with cigar smoke, he placed the cigar on the fire alarm in front of the laboratory entrance. You never know!

He loved photography, especially black and white photos, he was sporty and loved playing squash as well as downhill skiing, and he was very interested in art and proudly showed his original by Joseph Beuys to all his guests.

**Fig. 1:**
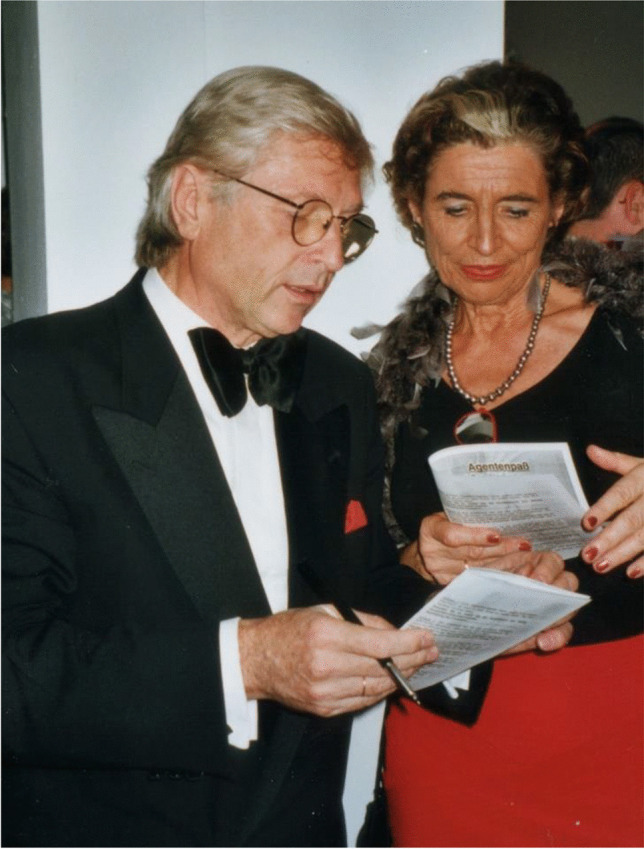
Stefan and Heidi Silbernagl, as we shall remember them. This photo shows the two as main actors in a James Bond competition. They present a mix of charm and sophistication, carried with a sense of effortless cool, while enjoying life

“Beauty will save the world,“ according to Fjodor Michailowitsch Dostojewski. Compared to the appearance of many other scientists at the various meetings, Stefan always added some unobtrusive elegance, not to say grandezza (Fig. 1). It was not the appearance of the “old” honorary physicians, but a light southern flair suitable to his red Alfa Romeo.

On January 20, 2025, Stefan Silbernagl died after a short, serious illness. This is a sad moment. But it is not sadness that should color our memory and remembrance. We will remember his open face, his heartly smile, and a haircut resembling Robert Redford. Adieu, mentor, colleague, and friend.

## Data Availability

No datasets were generated or analysed during the current study.
